# CD24 Expression and differential resistance to chemotherapy in triple-negative breast cancer

**DOI:** 10.18632/oncotarget.16203

**Published:** 2017-03-15

**Authors:** Xinyu Deng, Sophia Apple, Hong Zhao, Jeongyoon Song, Minna Lee, William Luo, Xiancheng Wu, Debra Chung, Richard J. Pietras, Helena R. Chang

**Affiliations:** ^1^ Gonda, UCLA Breast Cancer Research Laboratory and Revlon, UCLA Breast Center, Department of Surgery, David Geffen School of Medicine, University of California, Los Angeles, CA 90095-7028, USA; ^2^ Pathology and Laboratory Medicine, University of California, Los Angeles, CA 90095-1732, USA; ^3^ Department of Breast Surgery, First Affiliated Hospital of Zhejiang Chinese Medical University, Hangzhou, Zhejiang 310006, P. R. China; ^4^ Department of Surgery, East-West Medical Center, Kyung Hee University College of Medicine, Dongdaemun-gu, Seoul 02447, South Korea; ^5^ Department of Medicine, Division of Hematology-Oncology, David Geffen School of Medicine, University of California, Los Angeles, CA 90095-1678, USA

**Keywords:** breast cancer, CD24, drug resistance, EMT, chemotherapy

## Abstract

Breast cancer (BC) is a leading cause of cancer-related death in women. Adjuvant systemic chemotherapies are effective in reducing risks of recurrence and have contributed to reduced BC mortality. Although targeted adjuvant treatments determined by biomarkers for endocrine and HER2-directed therapies are largely successful, predicting clinical benefit from chemotherapy is more challenging. Drug resistance is a major reason for treatment failures. Efforts are ongoing to find biomarkers to select patients most likely to benefit from chemotherapy. Importantly, cell surface biomarkers CD44^+^/CD24^−^ are linked to drug resistance in some reports, yet underlying mechanisms are largely unknown. This study focused on the potential role of CD24 expression in resistance to either docetaxel or doxorubicin in part by the use of triple-negative BC (TNBC) tissue microarrays. *In vitro* assays were also done to assess changes in CD24 expression and differential drug susceptibility after chemotherapy. Further, mouse tumor xenograft studies were done to confirm *in vitro* findings. Overall, the results show that patients with CD24-positive TNBC had significantly worse overall survival and disease-free survival after taxane-based treatment. Also, *in vitro* cell studies show that CD44^+^/CD24^+/high^ cells are more resistant to docetaxel, while CD44^+^/CD24^−/low^ cells are resistant to doxorubicin. Both *in vitro* and *in vivo* studies show that cells with CD24-knockdown are more sensitive to docetaxel, while CD24-overexpressing cells are more sensitive to doxorubicin. Further, mechanistic studies indicate that Bcl-2 and TGF-βR1 signaling via ATM-NDRG2 pathways regulate CD24. Hence, CD24 may be a biomarker to select chemotherapeutics and a target to overcome TNBC drug resistance.

## INTRODUCTION

Breast cancer (BC) is a leading cause of cancer-related death in women worldwide [1]. Despite recent advances in targeted BC treatment, adjuvant chemotherapy remains the mainstay to improve survival in high-risk BC patients [2, 3]. Resistance to chemotherapy is a key factor in treatment failure. Better understanding of drug resistance mechanisms is crucial to advance BC management.

Triple-negative breast cancer (TNBC) is a BC subtype defined by lack of estrogen and progesterone receptors and absence of HER2 amplification [4]. As compared with other BC subtypes, TNBC patients have significantly worse prognosis despite use of chemotherapy [5]. Since there are no currently-approved targeted treatments for TNBC, chemotherapy is a standard intervention, yet patients often suffer early disease recurrence and metastasis due to development of drug resistance.

Cluster of differentiation 24 (CD24) is a small GPI-linked membrane glycoprotein with glycosylation sites that bind P-selectin [6]. As an adhesion molecule, CD24 is widely expressed in many cancer types, including renal, ovarian, lung and pancreatic cancers [7–9]. CD24 expression is also suggested to be a candidate marker for prognosis in breast cancer [10]. The CD44^+^/CD24^−^ phenotype is among the most widely studied biomarkers for breast cancer stem cells (CSC), but the clinical impact of these reported CSC biomarkers remains controversial [11, 12]. CD44^+^/CD24^−/low^ BC cells are reported to have tumor-initiating properties [12] and enhanced invasive activity [13]. Clinically, tumors with a higher fraction of CD44^+^/CD24^−^ cells are more commonly found in patients with distant metastases [14] and associate with poor clinical outcome [15]. Recent studies indicate TNBCs also have higher representation of CD44^+^/CD24^−^ cells [13, 15, 16], particularly among African American versus Caucasian women [17].

A cellular process that converts adherent epithelial cells into mesenchymal-like cells with the ability to migrate and invade adjacent tissues is termed epithelial-mesenchymal transition (EMT), and the reverse process is designated mesenchymal-epithelial transition (MET) [18]. Recently, breast cancer cells were reported to transit between EMT and MET states and to play different biologic roles during metastasis [19]. The biomarker set CD44^+^/CD24^−/low^ associates with more aggressive clinical-pathological features in BC [20]. CD44^+^/CD24^−/low^ cells generally display a mesenchymal phenotype [19, 21], while CD44+/CD24^+/high^ cells tend to associate with more differentiated epithelial features [22].

Previous reports indicate that CD24 is involved in EMT-MET transitions in breast cancer cells. However, the association of a CD44^+^/CD24^−/low^ population with the clinical outcome of patients with breast cancer and drug resistance, particularly to specific types of chemotherapeutics, is unclear [23]. Further, little is known about the regulatory mechanism for CD24 expression. Our earlier work indicated that treatment of BC cells with docetaxel induces the transition of BC cells from a CD44^+^/CD24^−/low^ phenotype to a CD44^+^/CD24^+/high^ phenotype that is more resistant to docetaxel [24]. Later, work by Goldman *et al*. supported our findings on such antitumor drug-induced phenotypic plasticity [25, 26]. In contrast, some studies suggest that CD44^+^/CD24^−/low^ cells are more resistant to chemotherapy, and that patients with tumors containing CD44^+^/CD24^−/low^ cells have worse survival [27, 28]. These contrasting data highlight the complexities in defining the role of CD24 in TNBC responses to therapy. In this work, we focused on *in vitro* and *in vivo* studies of CD24 expression and potential underlying signaling pathways in TNBC drug resistance.

## RESULTS

### Docetaxel and doxorubicin regulate CD24 expression in a different manner

**Figure 1:**
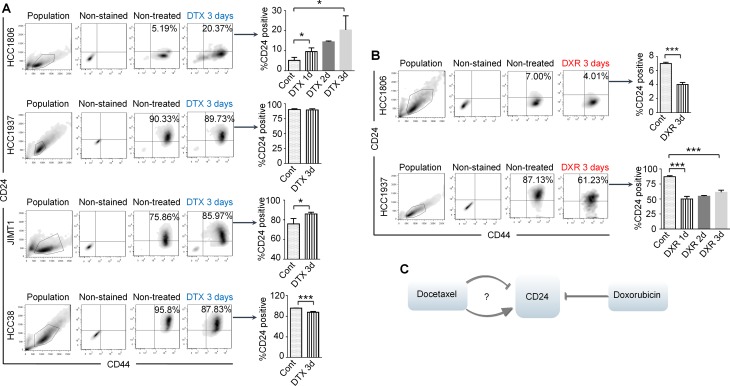
Docetaxel (DTX) induces CD24^+/high^ to CD24^−/low^, or CD24^−/low^ to CD24^+/high^ transitions or no change of CD24 expression in BC cell lines, while doxorubicin (DXR) only induces CD24^+/high^ to CD24^−/low^ transitions (**A**) and (**B**) show Fluorescence-Activated Cell Sorting (FACS) results and the respective bar graphs. Error bars represent standard error of the mean (SEM). The *p*-values were calculated using an unpaired *t* test. *P < 0.05; ***P < 0.001. (A) The cells were treated with 6 μM docetaxel for 1 to 3 days and then stained with CD24-PE and CD44-FITC for FACS analysis. HCC1806, HCC1937 and HCC38 are TNBC cell lines. JIMT-1 is a HER2-overexpressing BC cell line. (B) HCC1806 and HCC1937 were treated with 4 μM doxorubicin for 1 to 3 days and then stained with CD24-Brilliant Violet 421 and CD44-FITC. (**C**) The summarized results of A and B.

Drug-resistant cells are generally considered to be represented by cells that survive chemotherapy treatment. To study the relationship between CD24 functions and drug resistance, we treated cells with the two most commonly used drugs for TNBC, namely docetaxel and doxorubicin, and CD44/CD24 expression was tested before and after treatments. Overall, we investigated eight TNBC cell lines and compared these with six BC cell lines of other subtypes including three luminal and three HER2 positive cells, with representative results shown in Figure 1. In Supplementary Table 1, four of the eight TNBC cells had a main population of CD44^+^/CD24^−/low^ cells. Of the three HER2+ cell lines tested, only JIMT-1 had a large population of CD44^+^/CD24^−/low^, and the remaining were CD44^+^/CD24^+/high^. All luminal cell lines were CD44^+/−^/CD24^+/high^. The gating method and control information were described in Supplementary Figure 1.

After doxorubicin treatment, all CD44^+^/CD24^−/low^ cell lines remained unchanged, while CD44^+^/CD24^+/high^ cell lines showed decreased CD24 expression in the surviving cells (Figure 1; and data not shown). In contrast, cells responded differently after docetaxel treatment. All CD44^+^/CD24^−/low^ cell lines had increased CD24 expression after docetaxel treatment (Figure 1; and data not shown). Both HCC1937 and HCC38 cells are CD44^+^/CD24^+/high^; however, HCC1937 cells showed no change in CD24 expression, while HCC38 cells showed a decrease in CD24 expression after docetaxel treatment. Overall, our results suggest doxorubicin induces suppression of CD24 expression in CD44^+^/CD24^+/high^ cells, while docetaxel may decrease, increase or have no effect on CD24 expression in different cell lines. Our results also indicate that sensitivities of TNBC cells to the two drugs associate with the CD24 phenotype of surviving cells after drug treatments. The decreased CD24 expression in HCC1937 cells after doxorubicin and increased CD24 in HCC1806 cells after docetaxel as shown by FACS analyses were also confirmed by Western blot experiments (Supplementary Figure 2). Because CD24 expression in these cell lines changed rapidly after only a very short time of drug treatment before cell death occurred (Figure 5B, Supplementary Figure 2 and data not shown), the observed changes were less likely due to selective killing of particular cell populations.

To study whether expression of other important cancer biomarkers also change after chemotherapy treatment under the same experimental conditions, we tested CD133 and CD49f markers using FACS and Aldehyde Dehydrogenase (ALDH) activity using an established Aldefluor assay. In comparison with CD24 expression after the two drug treatments, ALDH, CD133 and CD49f displayed different patterns of expression with no clear correlates or trends identified. (Supplementary Figure 3 and data not shown)

### CD24 expression levels correlate with drug resistance

**Figure 2:**
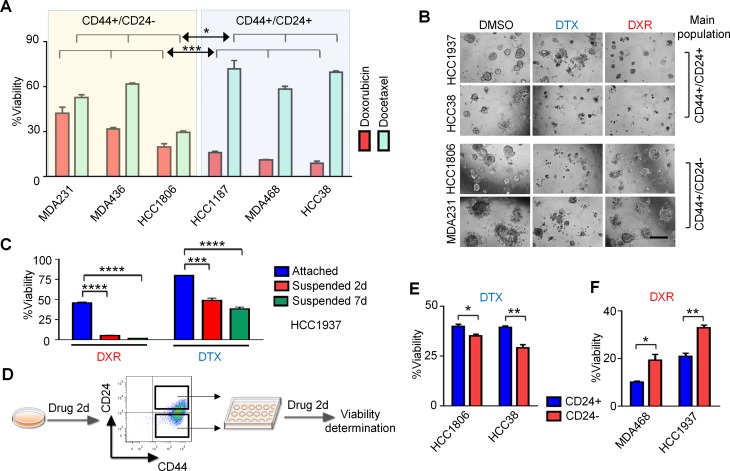
CD24^+/high^ TNBC cells are resistant to docetaxel (DTX) and CD24^−/low^ TNBC cells are resistant to doxorubicin (DXR) (**A**) The drug sensitivity assay results of six TNBC cell lines dosed with either 25.6 μM docetaxel or 6.4 μM doxorubicin are shown. *P*-values were calculated with Two-way ANOVA. *P < 0.05; ***P < 0.001. (**B**) After seeding in matrigel for 4 days, cells were treated with 0.6 μM docetaxel or 60 nM doxorubicin for 7 days. (**C**) HCC1937 cells were cultured under standard conditions or in ultralow-attachment plates for 2 days, then treated with 6 μΜ doxorubicin or 25.6 μΜ docetaxel for either 2- or 7 days. ***P < 0.001; ****P < 0.0001. (**D**) Work flow for experiments shown in (**E**) and (**F**). HCC1806 and HCC38 cells were treated with 25.6 μM docetaxel for 2-days. Treated cells were then sorted into CD44^+^/CD24^+/high^ and CD44^+^/CD24^−/low^ populations by FACS. Sorted populations were re-treated with 25.6 μM docetaxel, with cell viabilities determined after 2-days. MDA-MB-468 and HCC1937 cells underwent the same protocol except 6.4 μM doxorubicin was used. (E) and (F) *P*-values were calculated with unpaired *t* test. *P < 0.05; **P < 0.01. Error bars represent SEM. Scale bar, 200 μm.

To test the hypothesis that CD24 expression affects drug resistance, we performed drug sensitivity assays of different cell lines treated with either docetaxel or doxorubicin. The results show that cell lines with predominant populations of CD44^+^/CD24^+/high^ cells (HCC1187, MDA-MB-468 and HCC38) were more resistant to docetaxel, while cell lines with main populations of CD44^+^/CD24^−/low^ cells (MDA-MB-231, MDA-MB-436 and HCC1806) were more resistant to doxorubicin (Figure 2A). Similar results were found using drug treatment in 3D culture assays (Figure 2B). In suspension conditions, the CD24^+^ cell line HCC1937 became much more sensitive to doxorubicin (Figure 2C).

Because CD24 expression can be altered by drug treatment, we next determined the drug sensitivity of those cells remaining after chemotherapy treatment. As shown in Figure 2D, 2E and 2F, the new CD44^+^/CD24^−/low^ population induced by either docetaxel or doxorubicin was sorted out and found to be more resistant to doxorubicin compared with the CD44^+^/CD24^+/high^ population of the same cell lines, whereas the new CD44^+^/CD24^+/high^ population induced by docetaxel was more resistant to docetaxel compared with the CD44^+^/CD24^−/low^ population. In HCC38 cells, docetaxel sensitivity was improved in newly-induced CD44^+^/CD24^−/low^ cells. These results suggest- that CD44^+^/CD24^+/high^ cells were highly resistant to docetaxel, and CD44^+^/CD24^−/low^ cells were resistant to doxorubicin. Most of the cells became more resistant to the drugs after treatment in accord with the level of CD24 expression in surviving cells. Although HCC38 cells showed an induced population with decreased CD24 expression after docetaxel, the main population remained CD44^+^/CD24^+/high^, and continued to be docetaxel-resistant. These observations suggest that the drug resistance of tested cell lines is regulated by and/or associated with CD24 expression levels.

### Targeting CD24 or its inhibitor NDRG2 overcomes drug resistance

**Figure 3:**
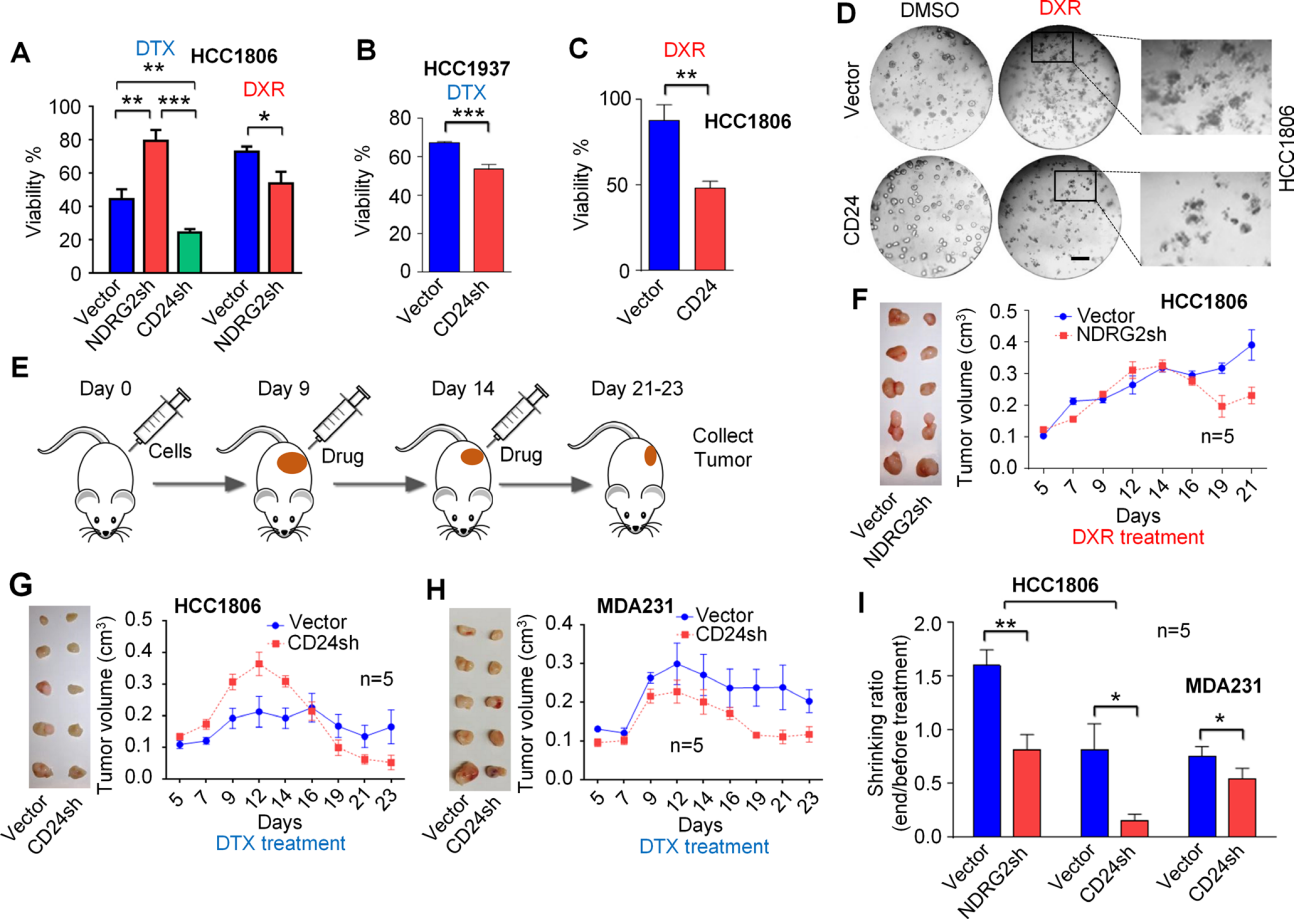
Targeting CD24 and its inhibitor gene NDRG2 by shRNAs improved chemotherapy sensitivity *in vitro* and *in vivo* (**A**, **B**, **C** and **D**) After seeding in matrigel for 5-days, HCC1806 cells were treated with 20 nM docetaxel (DTX) or 1.5 μM doxorubicin (DXR) for 4-days. HCC1937 cells were treated with 0.6 μM docetaxel. *P*-values of the compared groups were calculated using unpaired *t*-tests for cell total area. (**E**) These data show the working scheme for treatments used in tumor xenograft experiments. (**F**, **G**, **H**) These data show results of experiments with five tumors in each treatment group. (**I**) These data show statistical analyses of tumor shrinkage in the three pairs of experiments. *P*-values of each paired group were calculated with paired sample *t*-tests. *P < 0.05; **P < 0.01; ***P < 0.001. Error bars represent SEM. Scale bar, 200 μm.

To determine whether CD24 and potential CD24 downstream signaling regulate drug resistance, we used specific shRNAs *in vitro* to suppress expression of CD24 or the CD24 inhibitor NDRG2 [29]. After knockdown of CD24 in 3D culture, HCC1806 cells became more sensitive to docetaxel (Figure 3A). In contrast, cells with NDRG2 knockdown became more resistant to docetaxel and more sensitive to doxorubicin. Knockdown efficiency is shown in Supplementary Figure 2. Similar results were observed in another cell line, HCC1937, with knockdown of CD24 expression (Figure 3B). Conversely, overexpression of CD24 in HCC1806 leads to increased doxorubicin sensitivity (Figure 3C and 3D), a result consistent with enhancement of CD24 by suppression of the CD24 inhibitor NDRG2 as above.

To confirm our *in vitro* data, *in vivo* studies were conducted in a tumor xenograft model. As shown in Figure 3E and 3F, TNBC HCC1806 tumor xenografts with NDRG2 knockdowns showed marked tumor reduction after doxorubicin treatment as compared to controls. On the other hand, tumors of HCC1806 cells with CD24 knockdown demonstrated significant tumor reduction after docetaxel treatment as compared to controls (Figure 3G). Another TNBC line MDA-MB-231-with knockdown of CD24 also showed improved tumor response to docetaxel (Figure 3H) as predicted by the *in vitro* data. Statistical analysis of tumor shrinkage under different conditions as in Figure 3I demonstrate that inhibition of CD24 reduces docetaxel resistance, while increasing CD24 or suppressing CD24 inhibition improves doxorubicin sensitivity. These results suggest that targeting or modulating CD24 expression may overcome drug resistance in TNBC.

### TGF-βR1 and Bcl-2 regulate CD24 expression and drug sensitivities

**Figure 4:**
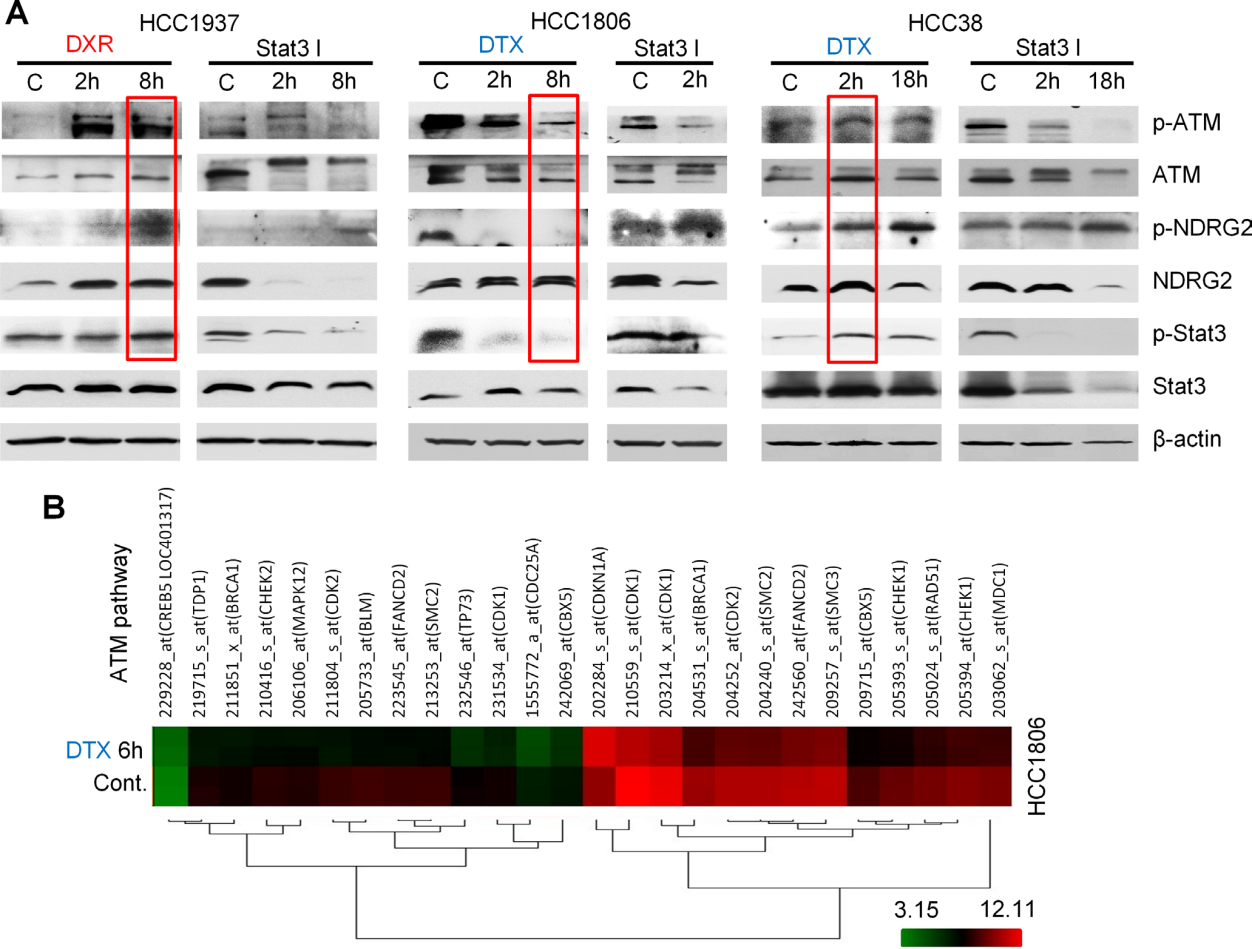
ATM signaling regulated by chemotherapy drugs (**A**) Results from three cell lines, HCC1937, HCC1806 and HCC38, treated with 6.4 μM docetaxel (DTX), 4 μM doxorubicin (DXR) or 5 μM STAT3 inhibitor VII. Levels of phospho-ATM, -NDRG2 and -STAT3 showed congruent changes following a similar trend: p-NDRG2 and p-STAT3 were increased when p-ATM was upregulated; p-NDRG2 and p-STAT3 were decreased when p-ATM was suppressed. (**B**) Gene microarray data show that expression of genes in the ATM pathway was dramatically reduced after 6 h of 4 μM docetaxel treatment. The genes changed ≥ 2 folds and *P* < 0.05.

**Figure 5:**
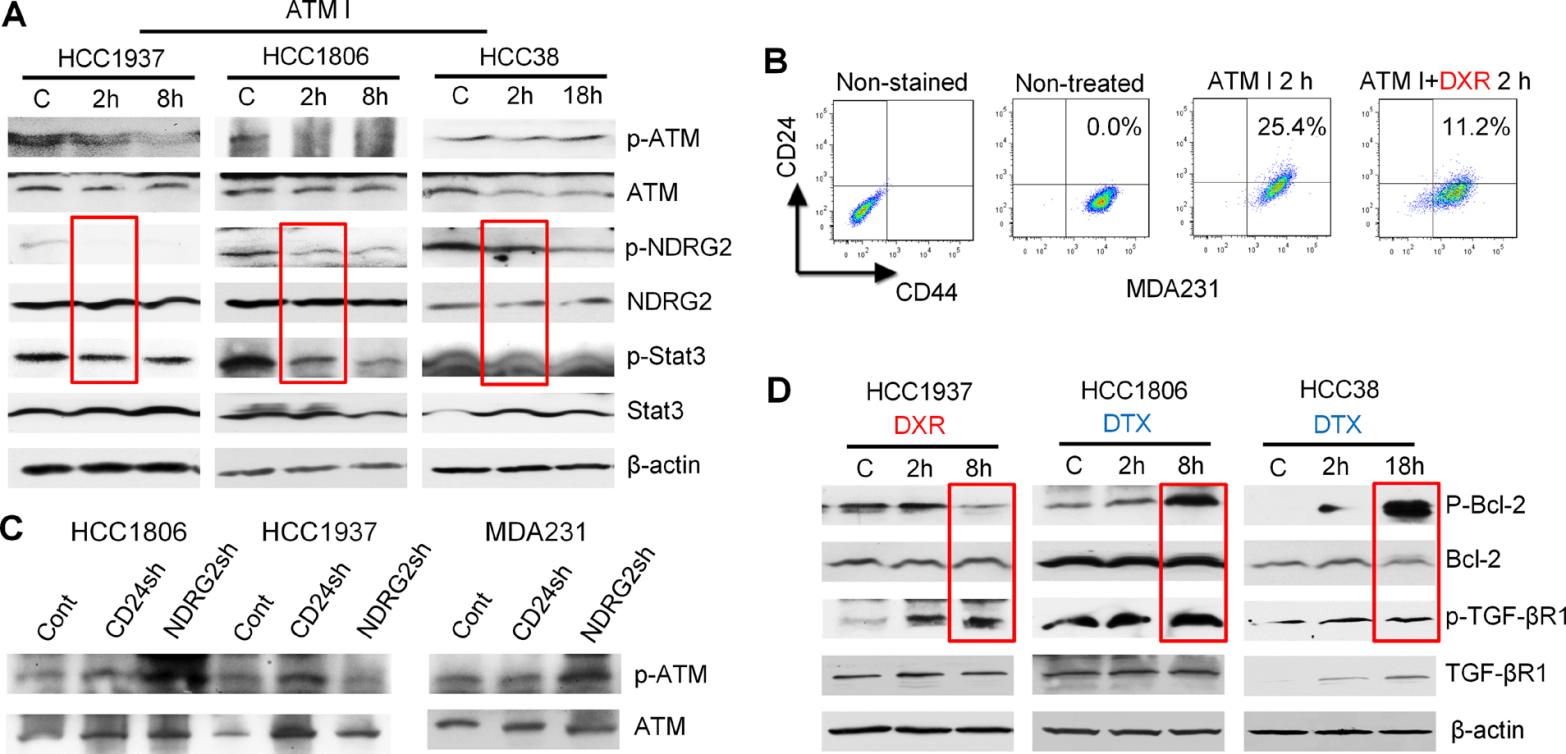
Bcl-2, TGF-βR1 and ATM signaling assessed by Western blot and FACS in selected TNBC cell lines (**A**) Cells were treated with 10 μM ATM inhibitor KU60019. (**B**) FACS results showed that 10 μM ATM inhibitor increased CD24 expression in MDA-MB-231 cells; and 4 μM doxorubicin reduced CD24 expression. (**C**) Selected cells were transfected with control vector, CD24 shRNA or NDRG2 shRNA. Knockdown of NDRG2 caused dramatic p-ATM increase in HCC1806 and MDA231 cells (**D**) The cells were treated with docetaxel 6.4 μM or doxorubicin 4 μM. Both docetaxel and doxorubicin increased p-TGF-βR1 in the three cell lines. Doxorubicin reduced p-Bcl-2 in HCC1937 and docetaxel elevated p-Bcl-2 in HCC1806 and HCC38.

**Figure 6:**
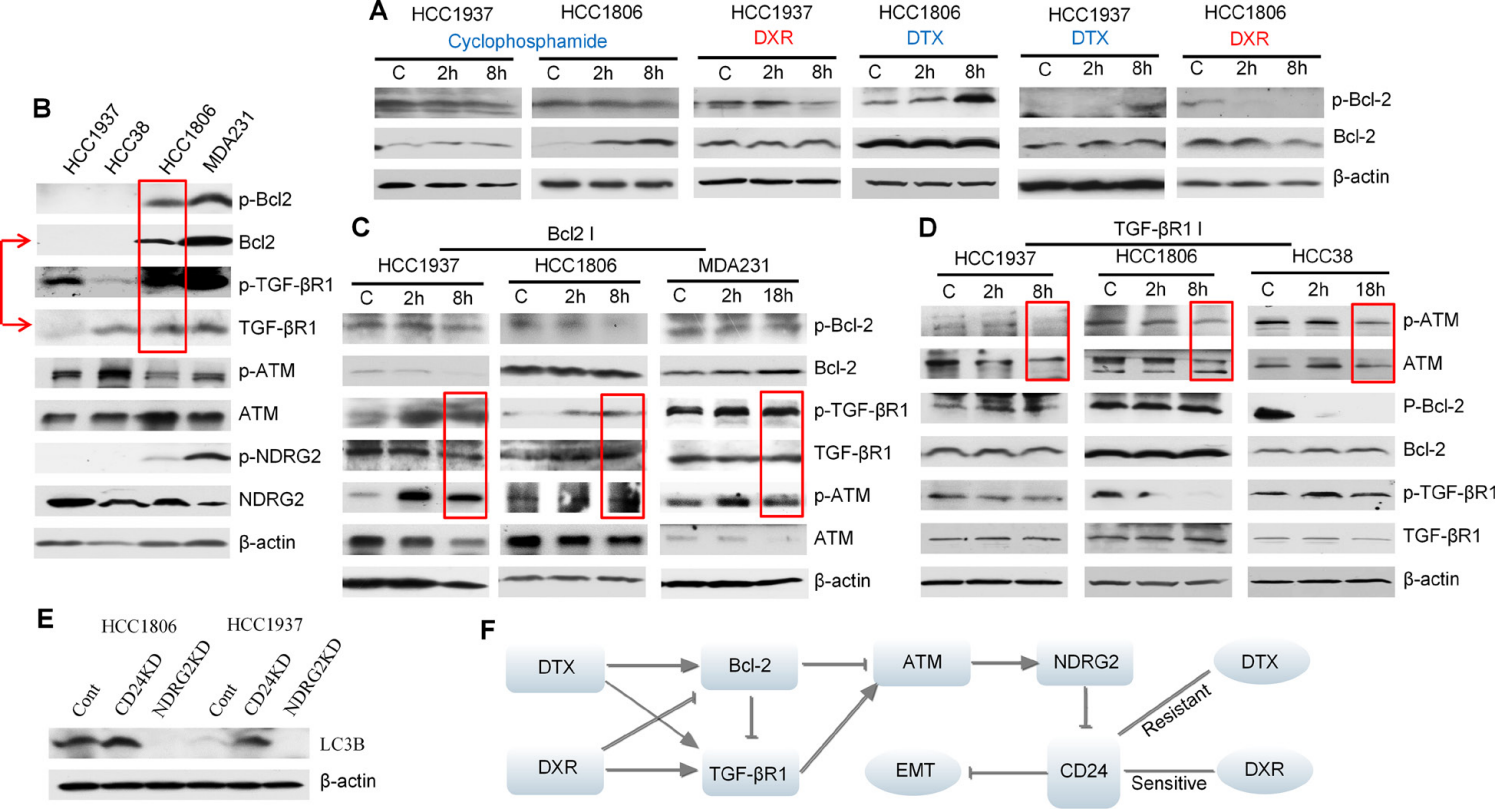
Interactions between Bcl-2, TGF-βR1 and ATM signaling in TNBC cells treated with either docetaxel (DTX) or doxorubicin (DRX) (**A**) and (**B**) Western blot results. Docetaxel: 6.4 μM; doxorubicin: 4 μM; cyclophosphamide: 2 μM. The results showed that doxorubicin reduced p-Bcl-2 in HCC1937 and HCC1806 and docetaxel increased p-Bcl-2 in the same cell lines. Cyclophosphamide had no significant effect on p-Bcl-2 in the two cell lines. HCC38 had a relatively higher basal level of TGF-βR1 compared to HCC1937. The bands of p-Bcl-2, Bcl-2 and β-actin in HCC1806 treated with DTX and HCC1937 treated with DXR have been shown in Figure 5. They were shown here again for a different comparison. (**C**) Cells were treated with 5 μM Bcl-2 inhibitor ABT-737. The results showed that Bcl-2 inhibitor stimulated p-TGF-βR1 and p-ATM, suggesting that Bcl-2 is an inhibitor of TGF-βR1 and ATM in the three cell lines. (**D**) Cells were treated with 5 μM TGF-βR1 inhibitor LY 364947. The results showed that TGF-βR1 inhibitor suppressed p-ATM and TGF-βR1 is stimulatory to p-ATM. (**E**) The Western blot results of autophagy marker-LC3B. The results showed that CD24 knockdown increased LC3B expression and NDRG2 knockdown eliminated LC3B expression. (**F**) Proposed diagram to summarize contrasting effects of doxorubicin and docetaxel on critical TNBC cell signaling pathways.

To investigate why docetaxel elicited variable CD24 expression in different BC cell lines, we focused on signaling pathways that could mediate observed phenomenon associated with CD24 expression. Of these, Bcl-2 was selected due to its reported involvement in paclitaxel resistance [30]. The CD44^+^/CD24^−/low^ phenotype is shown to be related to EMT [21], and our results show that HCC1806 cells undergo partial MET after docetaxel treatment; and MDA-MB-468 cells undergo EMT after doxorubicin treatment (Supplementary Figure 4). Since TGF-β is one of the most important inducers of EMT and is a secreted protein [31], TGF-βR1 is generally considered to reflect TGF-β downstream signaling [32]. Hence, TGF-βR1 was also selected for evaluation in the current study. We used gene microarrays to search for downstream signaling of Bcl-2 and TGF-β. The most differentially-expressed genes were listed in Supplementary Tables 2 and 3. Of interest, the ataxia-telangiectasia mutated (ATM) pathway was markedly inhibited in HCC1806 cells by treatment with docetaxel (Figure 4B; Supplementary Table 4) and was therefore assessed further. Previous reports showed that increased expression of Twist and reduced expression of CD24 contribute to EMT and to CSC self-renewal through activation of transcription 3 (STAT3)-dependent pathways in liver and breast cancers [33, 34]. As shown in Figure 4A, ATM signaling correlated positively with NDRG2 and STAT3 signaling as shown by simultaneous increase or decrease of p-ATM with p-NDRG2 and p-STAT3. Based on inhibitor treatments, our studies also show that ATM stimulates NDRG2, and increased NDRG2 negatively regulates ATM (Figure 5A and 5C). These results suggest ATM is upstream of NDRG2 and is inhibited by NDRG2 via negative feedback loops. Using a similar approach, we demonstrated that both TGF-βR1 and Bcl-2 were upstream of ATM with TGF-βR1 being stimulatory and Bcl-2 being inhibitory to ATM. Both docetaxel and doxorubicin stimulated TGF-βR1 signaling. For Bcl-2, docetaxel stimulated, while doxorubicin was suppressive; and cyclophosphamide had no effect (Figure 6). Our results also show that Bcl-2 expression in both CD44^+^/CD24^+/high^ cells -HCC1937 and HCC38- are very low. Further, HCC38 had higher levels of TGF-βR1 expression than HCC1937. Although CD44^+^/CD24^−/low^ cells (HCC1806 and MDA-MB-231) had high levels of TGF-βR1 activity, Bcl-2 activities in these cells were even more elevated thereby serving as a dominant signaling function (Figure 6B). The expression of autophagy marker-LC3B was next tested in CD24 knockdown and NDRG2 knockdown cell lines (Figure 6E). The results show that CD24 knockdown elevates LC3B expression and NDRG2 knockdown diminishes LC3B levels in the tested cells and suggest that CD24 might affect the autophagy process in these cells.

Taken together, our results indicate that interactions between Bcl-2/TGF-βR1 signaling regulate CD24-mediated drug resistance (Figure 6F). ATM stimulates NDRG2 activity which negatively regulates CD24. Doxorubicin may inhibit Bcl-2 and leave TGF-βR1 signaling unopposed, which leads to ATM stimulation and reduction of CD24 expression. In contrast, docetaxel may stimulate both TGF-βR1 and Bcl-2 signaling. Whether docetaxel stimulates or inhibits ATM appears to depend on the balance between activities of TGF-βR1 and Bcl-2 signaling. When Bcl-2 signaling dominates over TGF-βR1 signaling, ATM is inhibited, and vice-versa.

Molecular findings on the involvement of these key factors in drug resistance were also confirmed at the cellular level (Figure 5B; Supplementary Figures 5 and 6). Briefly, knockdown of Bcl-2 elicits a decrease of CD24 expression in HCC1937 and HCC1806 cells; knockdown of TGF-βR1 increases CD24 only in HCC1806 but not in HCC 1937 because of its high basal level. Knockdown of Bcl-2 also stimulates self-renewal of HCC1806 cells in suspension culture; while knockdown of TGF-βR1 or ATM kinase reduces self-renewal in the same cells. Also knockdown of Bcl-2 induces differentiation (3D sphere-like structures) of HCC1806 cells while knockdown of TGF-βR1 or ATM kinase decreases differentiation of these cells in 3D culture. The effects of TGF-βR1, Bcl-2 and ATM on cell drug sensitivities were confirmed using their corresponding inhibitors in HCC1806 and HCC1937 cells (Supplementary Figure 6E and 6F).

### CD24 is a potential biomarker for selection of chemotherapy regimens

**Figure 7:**
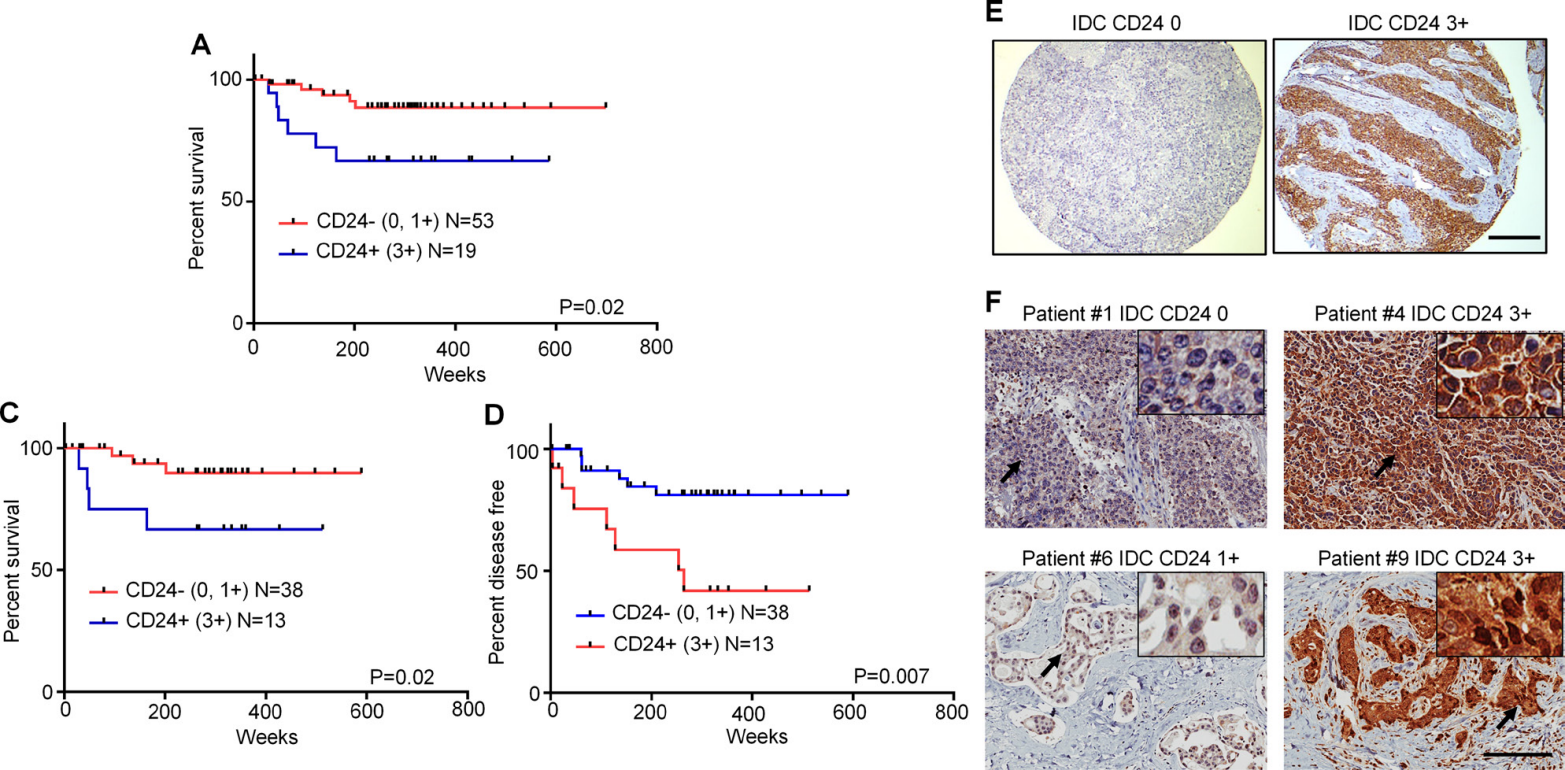
High expression of CD24 in archival tumor specimens from TNBC patients treated with taxane-based chemotherapy associate with poor prognosis Kaplan-Meier survival curves are shown in relation to CD24 expression in archival tumor specimens represented in a TNBC Microarray (TMA). (**A**) Overall survival analysis of all patients who received either doxorubicin- or taxane-based treatments. (**B**), (**C**) Data show that the survival analysis was limited to only patients who received taxane-based regimens. (B, overall survival analysis; C, disease-free survival analysis). (**D**) Representative CD24 IHC staining of TNBC TMA is shown. The specificity of CD24 staining was confirmed using TNBC cells stained with the same antibodies and IHC reagent kit (data not shown). (**E**, **F**) Representative CD24 staining of tumor samples from 4 of the 9 patients noted in Supplementary Table 5 is shown. Scale bar, 200 μm.

We next explored clinical significance of CD24 expression in archival patient specimens. For survival analysis, we selected 94 cases of TNBC patients who were treated with adjuvant chemotherapy. As a group, patients with CD24^−/low^ tumors had significantly better survival than those with CD24^+/high^ tumors (Figure 7A). Only 21 patients in the study cohort received anthracycline (doxorubicin) without taxanes (either paclitaxel or docetaxel). The better overall survival rate and disease free survival rate of patients with CD24^−/low^ tumors treated with taxane-based regimens was even more evidenced after excluding patients who received only doxorubicin (Figure 7B–7F). The relationship between CD24 expression and TNBC recurrence was further supported by studying a separate cohort of 9 cases shown in Supplementary Table 5. The results showed that all patients with high CD24 expression (2+ or 3+) before or after neoadjuvant treatment developed recurrent disease (mean time to recurrence 13.7 months) and all patients with low CD24 expression (0 or 1+) had no recurrent disease (mean follow up 47.5 months). Patient #5 whose tumor was 1+ before neoadjuvant treatment but increased to 2+ after taxane-based neoadjuvant treatment, later developed recurrent disease. Clinicopathological characteristics of patients whose tumors were included in the TNBC TMA are addressed in Supplementary Table 6.

## DISCUSSION

It is commonly reported that negative/low expression of CD24 is a characteristic biomarker of promoting tumor initiation and progression [12]. Results presented here demonstrated a unique mechanism of differential resistance to chemotherapeutics determined by CD24 and regulated in part by the balance of TGF-βR1 and Bcl-2 signaling. Our findings on tumor specimens from the clinic further supported that CD24 may be an important prognostic factor for TNBC patients who receive taxane-based treatment. In accord with our hypothesis, Chekhun *et al*. reported that TNBC patients with CD24^−/low^ tumors treated with doxorubicin-based chemotherapy regimens CAF (cyclophosphamide, doxorubicin and fluorouracil) or AC (doxorubicin and cyclophosphamide) had significantly worse survival rates than those patients with CD24^+/high^ tumors [28]. In addition, Lee et al. reported that patients treated with an AC regimen had a significantly shorter disease-free interval [35]. Independent preclinical findings also show that CD44^+^/CD24^−/low^ BC cell subpopulations exhibit resistance to anthracycline drugs [36, 37]. These several clinical and preclinical reports support our hypothesis that CD24^−/low^ BC cells are resistant to anthracycline treatment.

Our data also suggest that targeting CD24 and its regulatory pathways can reduce drug resistance in BC cells. When administering docetaxel to CD24^−/low^ tumors, adding agent(s) to block a phenotype switch from CD24^−/low^ to CD24^+/high^ may prevent development of adaptive resistance to docetaxel. Similarly, applying doxorubicin to CD24^+/high^ tumors, while blocking the transition from CD24^+/high^ to CD24^−/low^ phenotype, may improve doxorubicin sensitivity of treated cells. Future investigations in modulation of specific TNBC signaling pathways involved in regulating CD24 may lead to new therapeutic approaches. Our study further suggests that sequential administration of doxorubicin and taxane drugs may be more effective to improve TNBC treatment. We show that treating with docetaxel in CD24^−/low^ cells first, followed by doxorubicin, significantly improves the overall cytotoxic effect, whereas the reverse sequence of these drugs in the same cells results in a significantly reduced cytotoxicity (Supplementary Figure 6C). Of note, in CD24^+/high^ TNBC cells, the reverse sequence of drug administration (e.g., doxorubicin followed by docetaxel) yields better results (Supplementary Figure 6D).

The EMT process facilitates tumor cell migration through basement membrane, invasion into adjacent tissues, and penetration into the circulation [38, 39]. However, distant metastases from these tumors often show more differentiation with increased expression of luminal epithelial marker CD24 [40]. In addition, TNBCs with high CD24 expression associate with worse overall patient survival and shorter distant metastasis-free survival than tumors with low CD24 [23]. These observation may be related to our finding of high recurrence rates in patients with CD24^+^ TNBC who are treated with taxane-based regimens, but further investigation is needed to clarify the involvement of CD24 in metastasis as well as regulation of the host immune response [23, 41].

It is well-established that a major reason for treatment failure in patients with TNBC is development of resistance to chemotherapies which leads to early relapse or distant spread leading to patient death. Emerging reports suggest that metastatic tumor cells may evade cell death in part by the induction of autophagy, a tightly-regulated process involving the degradation of excessive, damaged or unnecessary proteins or injured organelles by lysosomal pathways to ensure the maintenance of cell viability [42, 43]. Several studies now suggest that autophagy plays an important role in cancer and in drug resistance [43–47]. Autophagy may promote cell survival in cancer cells treated with chemotherapy or lead to autophagic tumor cell death depending on the duration or extent of the induction of this process. As noted in our work, the potential relationship between autophagy and chemotherapeutic drugs is being explored in the context of the regulation of autophagy by CD24 which is modulated, in turn, by Bcl-2, ATM and associated signaling pathways [46, 47]. Our studies using antisense tools to up-regulate and down-regulate CD24 in breast cancer cells suggest a relationship between CD24 expression and biomarkers of autophagy *in vitro*. Whether autophagy is largely pro-tumorigenic or anti-tumorigenic is unclear, but independent efforts are underway to investigate autophagy as a potential target of antitumor drugs and the targeting of autophagy as a therapeutic strategy to modulate drug resistance [43–47]. This work helps to contribute to that effort [27, 45–47]. Further, the induction of autophagy in cancer cells is also associated with inhibition of cell lysis by cytotoxic T-lymphocytes, suggesting that regulation of autophagy may be important in the future development of immunotherapeutic strategies to block immune escape by TNBC [48].

Our findings to date suggest that regulation of CD24 expression in tumor cells is due in part to the balance of Bcl-2 and TGF-βR1 signaling via downstream activities of ATM and NDRG2, a notable tumor suppressor gene product that regulates CD24 expression to decrease the metastatic potential of breast cancer cells. Independent reports concur with several aspects of our findings such as the induction of ATM by doxorubicin in cardiac myocytes [49] and TGFβ- induced apoptosis via ATM in epithelial cells [50]. In addition, docetaxel is reported to enhance B1/Cdk1-mediated phosphorylation of Bcl-2 [51], while Hao *et al*. report that down-regulation of Bcl-2 enhances the sensitivity of polyploid cells to docetaxel [52]. How the downstream signaling of CD24 causes the change in sensitivity of tumor cells to taxane versus anthracylines remains to be answered. Since autophagy was previously reported to related closely to the CD44^+^/CD24^−^ status in TNBC cells [53], we assessed the autophagy biomarker LC3B in cells with selective knockdown of CD24 expression or in those with suppression of the CD24 inhibitor NDRG2. The results show that knockdown of CD24 led to a significant increase of autophagy marker LC3B, while knockdown of NDRG2 reduced LC3B in TNBC cell lines HCC1806 and HCC1937 (see Figure 6E). Importantly, independent reports also show that enhanced autophagy in BT-549 and MDA-MB-231 cells helped to promote tumor cell survival after doxorubicin treatment [54]. Also consistent with our findings was the report that diminished levels of autophagy-initiating genes caused MCF-7 and SKBR-3 cells to develop resistance to taxane therapy [55]. Taken together with independent research, this evidence suggests the hypothesis that selective changes in CD24 expression may regulate the drug sensitivities of tumor cells in part through modulation of cellular autophagy status. Further, these data on the identification of specific downstream pathways that regulate CD24 may help to explain prior controversial findings regarding the role of CD24 in drug resistance and phenotypic plasticity in TNBC cells.

To further understand the mechanism of CD24-regulated drug sensitivity in TNBC, we also assessed other cancer stem cell markers, such as ALDH, CD133 and CD49f. Interestingly, changes in the patterns of expression of these other cancer stem cell markers were different from those of CD24 expression. There was no clear trend of changes observed among different cell lines after the two drug treatments. Thus, more studies are needed in the future to define the potential relationships of other cancer stem cell markers with drug resistance in breast cancers.

In summary, the current data suggest that CD24 is a promising biomarker candidate to guide chemotherapy selection in the clinic, particularly in choosing between commonly-used TNBC therapeutics such as docetaxel and doxorubicin. This strategy may potentially be applied in future clinical trials and, if successful, could help to improve chemotherapy selection for the benefit of TNBC patients.

## MATERIALS AND METHODS

### Cell lines and cell culture

All cell lines except those noted below were obtained from the American Type Culture Collection (Manassas, VA, U.S.A.) and were used within six months of receipt for experimentation. JIMT-1, HCC1419 and SKBR3 cells were provided by Dr. Dennis Slamon (UCLA) and were authenticated in his laboratory by confirmation of genomic DNA, in comparison with the ATCC database. Experiments using these cell lines were also done within six months of receipt. Cells were maintained in Dulbecco's Minimal Essential Medium (Invitrogen, Carlsbad, CA, U.S.A.) or RPMI 1640 (Invitrogen) with 10% heat-inactivated Fetal Bovine Serum (FBS; Fisher Scientific, Pittsburgh, PA, U.S.A.), 100 units/mL penicillin, and 100 μg/mL streptomycin, at 37°C in 5% CO_2_. To assay self-renewal capacity, cells were seeded in Corning Ultra-Low Attachment 96-well Plates (Fisher Scientific) at 10,000 cells/well and cultured two days [56]. For 3D culture, cells were seeded at 10,000 cells/well in 96-well plates and grown in 40 μl Matrigel using established methods (Fisher Scientific) [57].

### Transfection

The siRNA against Bcl-2 (Cat No. AM16708), TGF-βR1 siRNA (Cat No. AM51331), and control siRNA (Cat No. AM4611) were from Invitrogen. Transfection of siRNAs was done with Lipofectamine^®^ RNAiMAX Transfection Reagent (Invitrogen) according to manufacturer's instructions. Cells were collected for analysis at 48 h post-transfection.

For plasmid transfections, HCC1806 cells were transfected with human CD24 with pCMV6-AC-GFP vector (Cat No. RG209542) or negative control (Cat No. PS100010) (OriGene Technologies, Rockville, MD, U.S.A.) using Lipofectamine 2000 (Invitrogen) according to manufacturer's instructions. Cells were sorted for GFP-positive markers 2–5 times to select transfected cells with stable staining.

To suppress CD24 and NDRG2 expression, HCC1937, HCC1806 and MDA-MB-231 cells were transfected with either CD24 shRNA (Cat No. TF321436), NDRG2 shRNA (Cat No. TF303005) or negative control vector (OriGene Technologies) using Lipofectamine 2000 (Invitrogen) according to manufacturer's instructions. Cells were sorted for RFP-positivity 2 -5 times to obtain transfected cells with stable staining.

### Fluorescence-activated cell sorting (FACS) and aldefluor assay

CD24-PE (ML5 clone) and CD44-FITC antibodies were from BD Biosciences (San Jose, CA, U.S.A.). To evade the autofluorescence of doxorubicin, Brilliant Violet 421™ anti-human CD24 Antibody (ML5 clone, BioLegend, San Diego, CA, U.S.A.) was used for doxorubicin-treated cells and the related control cells. For each sample, triplicates of 2 × 10^5^ cells/well were cultured in 6-well culture plates and treated with selected reagents. After treatment, cells were detached from plates using StemPro^®^ Accutase^®^ Cell Dissociation Reagent (Invitrogen), followed by centrifugation and re-suspension in 100 μl cold FACS buffer I (growth medium/PBS, 1:1, vol/vol). Cells were then transferred to 96-well U-bottom plates (Genesee Scientific, San Diego, CA, U.S.A.). Cells were incubated with antibodies on ice 30 min, spun down and washed 3-times with FACS buffer I. Cells were then re-suspended with FACS buffer II (5% FBS in PBS with 2 mM EDTA) and submitted for analysis or cell sorting. The same numbers of cells were analyzed in the same set of experiments. ALDH activity was tested using an ALDEFLUOR^™^ Kit (STEMCELL Technologies Inc., Vancouver, BC, Canada) according to the manufacturer's instructions. Briefly, single cells were suspended in ALDH assay buffer containing ALDH substrate BODIPY-aminoacetaldehyde (BAAA) and incubated at 37°C for 40 min. The specific ALDH inhibitor diethylaminobenzaldehyde at 50 mM was used as a negative control. Stained cells were analyzed by flow cytometry. Results were analyzed by FlowJo 7.6 (FlowJo LLC, Ashland, OR, U.S.A.).

### Drug sensitivity assay

Cells were seeded at a density of 10,000/well in 96-well plates (Fisher Scientific) and then treated with selected drugs or DMSO negative control for 48 h. Docetaxel, doxorubicin hydrochloride and cyclophosphamide monohydrate were from Sigma-Aldrich (St. Louis, MO, U.S.A.). At the end of treatment, cell viabilities were determined by using CellTiter-Glo Luminescent Cell Viability Assays (Promega, Madison, WI, U.S.A.). All experiments were performed in triplicate.

### Immunofluorescence staining and microscopy

Mouse anti-E-cadherin antibody was from Invitrogen, and mouse anti-vimentin antibody was from Abcam (Cambridge, MA, U.S.A.). Secondary antibodies were from Invitrogen. Approximately 2.5 × 10^4^ cells were seeded on a 4-well Lab-TekII Chamber Slide and treated with DMSO or chemotherapeutics. After 48 h, cells were washed twice with PBS and incubated with blocking solution (10% goat serum in PBS). Following three washes with PBS, cells were incubated with primary antibodies at 4°C for 100 min and were followed by 3- washes with PBS for 15 min. Cells were finally incubated with secondary color-conjugated antibodies (Invitrogen) for 100 min. Slides were washed thoroughly with PBS and mounted with ProLong^®^ Gold Antifade Reagent (Thermo Fisher Scientific Inc.). All matched samples were photographed (control and test) using a Nikon fluorescence microscope with identical exposure times.

### Quantitative RT-PCR

Quantitative real-time PCR was performed using glyceraldehyde-3-phosphate dehydrogenase (GAPDH) as a housekeeping reference and EXPRESS One-Step SYBR^®^ GreenER™ (Thermo Fisher Scientific Inc.) on an Applied Biosystems 7500 Fast Real-Time PCR system (Thermo Fisher Scientific Inc.), using the standard protocol as recommended by the manufacturer. The condition used for cDNA synthesis was 50°C for 5 min, and the condition for elongation was 60°C for 1 min. The primer sequences listed below were designed as reported before [58]:

hGAPDH-5 ACCCAGAAGACTGTGGATGG

hGAPDH-3 TCTAGACGGCAGGTCAGGTC

hNcad-5 ACAGTGGCCACCTACAAAGG

hNcad-3 CCGAGATGGGGTTGATAATG

hSnail-5 CCTCCCTGTCAGATGAGGAC

hSnail-3 CCAGGCTGAGGTATTCCTTG

hTwist-5 GGAGTCCGCAGTCTTACGAG

hTwist-3 TCTGGAGGACCTGGTAGAGG

hSlug-5 GGGGAGAAGCCTTTTTCTTG

hSlug-3 TCCTCATGTTTGTGCAGGAG

### Gene microarray

HCC1806 cells were treated with DMSO or 4 μM docetaxel for 6 h. After treatment, mRNAs of samples were prepared and submitted for Affymetrix Human Genome U133 Plus 2.0 Array (Affymetrix, Santa Clara, CA, U.S.A.) analysis in duplicate. Total RNA was extracted using TRIzol Reagent (Life Technologies, NY, U.S.A.) followed by Qiagen column purification (Qiagen, Valencia, CA, U.S.A.). The array hybridizations were performed by the UCLA Clinical Microarray Core Service following standard Affymetrix GeneChip Expression Analysis protocols. Acquisition of the array image was undertaken using Affymetrix GeneChip Command Console 4.0 (Affymetrix). Subsequent raw data were analyzed using Partek Genomics Suite 6.4 (Partek, St. Louis, Missouri, U.S.A.) and Affymetrix Transcriptome Analysis Console (Affymetrix, Version 2.0.0.9). We used the RMA algorithm for data normalization. Global functional analyses, network analyses and canonical pathway analyses were performed using Ingenuity Pathway Analysis (Ingenuity^®^ Systems, www.ingenuity.com). Data obtained from the microarray are deposited at Gene Expression Omnibus (http://www.ncbi.nlm.nih.gov/geo/query/acc.cgi?acc=GSE70690).

### Western immunoblot methods, inhibitors and antibodies

Cells were lysed in RIPA buffer supplemented with Phosphatase Inhibitor Cocktail Tablets and Protease Inhibitor Cocktail Tablets (Roche, Basel, Switzerland). For signaling analyses, cells seeded onto 6-well plates at 2 × 10^5^ cells/well were treated with selected chemotherapeutics or inhibitors. TGF-βR1 inhibitor LY364947 was purchased from Cayman Chemical (Ann Arbor, Michigan, U.S.A.) and LY2157299 was from Selleckchem (Houston, TX, U.S.A.). Stat3 Inhibitor VII was from EMD Millipore (Billerica, MA, U.S.A.). ATM kinase inhibitor KU60019 was from Santa Cruz Biotechnology (Dallas, TX, U.S.A.). Bcl-2 inhibitor ABT-737 was from Selleckchem and G3139 was from Alpha DNA (Montreal, Quebec, Canada). These inhibitors were selected based on prior reports [59–62]. Sheep anti-NDRG2 phospho-Thr348 antibody was from Kinasource Limited (Dundee, Tayside, UK). Rabbit anti-TGF-βR1 phospho S165 antibody was from Abcam. Rabbit anti-TGF-βR1 antibody, mouse anti-Bcl-2 antibody and mouse anti-Stat3 antibody were purchased from EMD Millipore. Mouse anti-human CD24 antibody (ML5 clone) and mouse anti-Stat3 Phospho (Tyr705) antibody were from BioLegend. Rabbit anti-NDRG2 antibody, rabbit anti-ATM antibody, mouse anti-Phospho-ATM (Ser1981) antibody, rabbit anti-Phospho-Bcl-2 (Ser70) antibody, anti-mouse IgG HRP-linked antibody and anti-rabbit IgG HRP-linked antibody were from Cell Signaling (Danvers, MA, U.S.A.). Donkey anti-Sheep IgG HRP-linked antibody was purchased from Invitrogen. Mouse anti-β-actin antibody was from Sigma-Aldrich. Western blotting was done by separating equal amounts of proteins on SDS-PAGE and transferring samples onto nitrocellulose membranes prior to detection with indicated antibodies and SuperSignal West Pico Chemiluminescent Substrate and SuperSignal ELISA Femto Maximum Sensitivity Substrate (Fisher Scientific). β-actin was used as loading control. All experiments were performed at least twice.

### Mice and tumor xenograft experiments

CBySmn.CB17-Prkdc^scid^/J 4- to 6-week-old female mice from The Jackson Laboratory (Bar Harbor, Maine, U.S.A.) were used in this study. The protocol was approved by UCLA's Office of Animal Research Oversight (OARO) and the Institutional Animal Care and Use Committee (IACUC), with procedures performed under supervision of UCLA's Division of Laboratory Animal Medicine. Mice were housed in aseptic conditions in micro-isolator cages and were provided sterile food and water.

Tumor cells were adjusted to a concentration of 3 × 10^6^ in 50 μl growth medium mixed with 50 μl matrigel. 100 μl cell suspension was injected subcutaneously using a 25G sterile needle in the 4^th^ mammary fat pad of each mouse under isoflurane anesthesia. Mice were then assigned based on tumor size after *one week* and divided into 6 treatment groups with 5 mice/ group. Groups 1 and 2 were injected with HCC1806 cells transfected with vector control or NDRG2 shRNA. Groups 3 and 4 were injected with HCC1806 cells transfected with vector control or CD24 shRNA. Groups 5 and 6 were injected with MDA-MB-231 cells transfected with vector control or CD24 shRNA. Chemotherapy drugs were administered intraperitoneally on days 9 and 14 after tumor inoculation. Groups 1 and 2 were treated with doxorubicin at 0.06 mg/120 μl/mouse and Groups 3 to 6 were treated with docetaxel at 0.28 mg/120 μl/ mouse. Tumors were measured at specified time points, with tumor volume calculated as follows: tumor volume = ½(width)^2^ × (length). At the end of experiments, mice were euthanized by CO2 inhalation at 21 or 23 days after tumor injection, and tumors were harvested.

### Tissue microarrays (TMA), regular tumor tissue samples and corresponding clinical and pathological database

A cohort of 94 TNBC specimens from the UCLA Pathology Tumor Bank were used for tissue microarray construction under IRB-approved protocols (#11-003372). Clinical-pathological characteristics, treatments, and outcomes of the 94 patients were abstracted from an IRB-approved retrospective database at the Revlon/UCLA Breast Center (#11-000826).

To further confirm the relationship between CD24 expression and TNBC recurrence, we studied additional 9 patients treated with taxane-based chemotherapy (neoadjuvant or/and adjuvant). Tumor samples from these 9 cases were stained for CD24 by a laboratory researcher and read by a breast pathologist. Both investigators were blinded for clinical information. The standard IHC staining protocol is described below.

### Immunohistochemical analysis of CD24

After antigen retrieval in Dako Cytomation Target Retrieval Solution (DAKO, Carpinteria, CA, U.S.A.), slides were stained with primary antibodies (CD24, ML5 clone) and biotin-conjugated secondary antibodies, followed by incubation with Streptavidin Peroxidasee and Substrate-Chromogen solution (DAKO). Blinded immunohistochemical scoring was performed by a breast pathologist. CD24 staining was detected at the membrane and cytoplasm of tumor cells, and scoring was done as follows: 0, 0–10% of positive tumor cells; 1+, 10–25% of positive tumor cells; 2+, 25–50% of positive tumor cells; 3+, more than 50% of positive tumor cells. The cases were divided into negative/low, if scored 0 or 1+, and significantly positive cases, when scored as 3+. Patients initially presenting with metastases of any type, local or regional recurrence, or another primary cancer were excluded (*n* = 10) from the survival analysis.

### Statistics

All tests were two-sided, and *p*-values < 0.05 were considered statistically significant. Statistical calculations were done with Graphpad Prism 6 (GraphPad Software, Inc., La Jolla, U.S.A.) and Excel (Microsoft, Redmond, WA, U.S.A.). Individual statistical methods are described in appropriate figure legends and other methods sections.

## SUPPLEMENTARY MATERIALS FIGURES AND TABLES


