# Supplementary material for: CD24 Expression and differential resistance to chemotherapy in triple-negative breast cancer
Source: Oncotarget. 2017 Mar 15;8(24):38294–308. doi: 10.18632/oncotarget.16203 (PMC5503533; doi:10.18632/oncotarget.16203)
Supplement: Supplementary file 1 [file oncotarget-08-38294-s001.pdf]

CD24 Expression and differential resistance to chemotherapy in triple-negative breast cancer

Supplementary Materials

Table 1: The percentages of CD44+/CD24 ± cell populations of breast cancer cell lines

Cell line	Subtype	Classification	% CD44+/CD24+*	% CD44+/CD24–*
HCC1187	ER– PR– HER-2–	TNBC	93.93 ± 0.12	5.59 ± 0.12
HCC1937	ER– PR– HER-2–	TNBC	72.07 ± 0.74	27.80 ± 0.75
MDA-MB-468	ER– PR– HER-2–	TNBC	98.47 ± 0.50	1.34 ± 0.50
HCC38	ER– PR– HER-2–	TNBC	95.73 ± 0.15	4.20 ± 0.16
HCC1806	ER– PR– HER-2–	TNBC	5.19 ± 0.86	93.53 ± 0.81
MDA-MB-436	ER– PR– HER-2–	TNBC	6.44 ± 0.41	93.30 ± 0.35
MDA-MB-157	ER– PR– HER-2–	TNBC	37.23 ± 6.43	62.53 ± 6.40
MDA-MB-231	ER– PR– HER-2–	TNBC	7.77 ± 0.39	92.03 ± 0.45
JIMT-1	ER– PR– HER2+	HER2	72.33 ± 3.57	27.40 ± 3.60
HCC1419#	ER– PR– HER2+	HER2	5.74 ± 0.53	0
SKBR3	ER– PR– HER2+	HER2	99.53 ± 0.20	0.04 ± 0.04
T47D	ER+ PR+/– HER-2–	Luminal A	90.23 ± 3.70	1.75 ± 1.24
MCF-7	ER+ PR+/– HER2–/Low	Luminal A	27.37 ± 0.48	1.38 ± 0.02
BT-474	ER– PR+ HER2+	Luminal B	93.33 ± 1.95	0.52 ± 0.51

Cells were stained with CD24 and CD44 antibodies and the conjugated colors were detected by FACS. *, Mean ± SEM. #, the main population of H1419 is CD44–.

Supplementary Table 2: Gene microarray results

	Transcript Cluster ID	Gene Symbol	ANOVA *p*-value	Fold Change (linear)
1	210873_x_at	APOBEC3A<br>APOBEC3A_B	0.004	14.59
2	214920_at	THSD7A	< 0.001	13.6
3	213894_at	THSD7A	< 0.001	12.86
4	226535_at	ITGB6	< 0.001	11.2
5	225987_at	STEAP4	0.004	9.39
6	217109_at	MUC4	0.001	9.34
7	213796_at	SPRR1A	0.001	9.22
8	217110_s_at	MUC4	0.007	9.09
9	241703_at	RUNDC3B	< 0.001	8.11
10	210665_at	TFPI	0.007	8.1
11	207526_s_at	IL1RL1	< 0.001	8.05
12	213258_at	TFPI	< 0.001	7.79
13	205532_s_at	CDH6	< 0.001	7.65
14	230008_at	THSD7A	< 0.001	7.48
15	202768_at	FOSB	0.001	7.41
16	205306_x_at	KMO	0.009	6.59
17	215321_at	RUNDC3B	0.004	6.58
18	205533_s_at	CDH6	0.02	6.45
19	208083_s_at	ITGB6	< 0.001	6.41
20	205959_at	MMP13	0.004	6.36
21	229159_at	THSD7A	0.009	6.27
22	225673_at	MYADM	< 0.001	6.13
23	211138_s_at	KMO	0.001	6.1
24	210664_s_at	TFPI	< 0.001	6
25	219909_at	MMP28	0.009	5.93
26	205681_at	BCL2A1	0.005	5.9
27	227314_at	ITGA2	< 0.001	5.78
28	1554062_at	XG	< 0.001	5.71
29	201860_s_at	PLAT	< 0.001	5.64
30	219529_at	CLIC3	< 0.001	5.6
31	211548_s_at	HPGD	< 0.001	5.53
32	218468_s_at	GREM1	< 0.001	5.53
33	227759_at	PCSK9	< 0.001	5.5
34	203914_x_at	HPGD	< 0.001	5.39
35	224941_at	PAPPA	0.003	5.37
36	203913_s_at	HPGD	< 0.001	5.31
37	210397_at	DEFB1	< 0.001	5.3
38	214803_at	CDH6	0.001	5.27
39	217127_at	CTH	< 0.001	5.24
40	202672_s_at	ATF3	< 0.001	5.15
41	223278_at	GJB2	< 0.001	5.05
42	201626_at	INSIG1	0.002	4.98
43	215034_s_at	TM4SF1	< 0.001	4.92
44	231941_s_at	MUC20	< 0.001	4.89
45	236001_at	LINC00675	< 0.001	4.89
46	226847_at	FST	< 0.001	4.85
47	204895_x_at	MUC4	0.008	4.83
48	228128_x_at	PAPPA	< 0.001	4.81
49	231779_at	IRAK2	0.005	4.76
50	204446_s_at	ALOX5	< 0.001	4.71

The top 50 genes significantly up-regulated by docetaxel when compared with DMSO control in HCC1806.

Supplementary Table 3: Gene microarray results

	Transcript Cluster ID	Gene Symbol	ANOVA *p*-value	Fold Change (linear)
1	220115_s_at	CDH10	0.006	–7.34
2	213906_at	MYBL1	0.002	–6.61
3	1556300_s_at	SIM1	0.002	–6.42
4	238029_s_at	SLC16A14	< 0.001	–5.27
5	1557129_a_at	FAM111B	< 0.001	–4.73
6	228038_at	SOX2	0.003	–4.7
7	220466_at	CCDC15	0.005	–4.63
8	205051_s_at	KIT	0.002	–4.44
9	1557385_at	FAM161A	0.002	–4.3
10	204126_s_at	CDC45	< 0.001	–4.24
11	241360_at	CCDC15	0.004	–4.22
12	1557217_a_at	FANCB	0.005	–4.22
13	1552680_a_at	CASC5	0.001	–4.14
14	223700_at	MND1	< 0.001	–4.13
15	219368_at	NAP1L2	< 0.001	–4.12
16	218806_s_at	VAV3	0.03	–4.08
17	229901_at	ZNF488	0.005	–4.04
18	216228_s_at	WDHD1	< 0.001	–4
19	204159_at	CDKN2C	0.002	–3.97
20	1557218_s_at	FANCB	< 0.001	–3.97
21	204441_s_at	POLA2	< 0.001	–3.92
22	205053_at	PRIM1	< 0.001	–3.92
23	219703_at	MNS1	0.001	–3.88
24	220840_s_at	C1orf112	0.002	–3.86
25	1553244_at	FANCB	0.003	–3.85
26	223599_at	TRIM6	0.008	–3.82
27	227713_at	KATNAL1	< 0.001	–3.81
28	211814_s_at	CCNE2	0.003	–3.74
29	223274_at	TCF19	< 0.001	–3.7
30	1557128_at	FAM111B	0.002	–3.67
31	223790_at	KATNAL1	< 0.001	–3.65
32	243840_at	CLSPN	0.001	–3.65
33	204603_at	EXO1	0.004	–3.64
34	204727_at	WDHD1	< 0.001	–3.62
35	213761_at	MDM1	< 0.001	–3.61
36	227349_at	HELLS	0.001	–3.61
37	205280_at	GLRB	0.005	–3.6
38	204728_s_at	WDHD1	0.003	–3.57
39	205909_at	POLE2	< 0.001	–3.57
40	221703_at	BRIP1	0.02	–3.57
41	203967_at	CDC6	< 0.001	–3.55
42	206382_s_at	BDNF	0.007	–3.55
43	203276_at	LMNB1	< 0.001	–3.54
44	219494_at	RAD54B	< 0.001	–3.54
45	1553120_at	CLSPN	0.01	–3.54
46	211124_s_at	KITLG	< 0.001	–3.51
47	225975_at	PCDH18	< 0.001	–3.5
48	209891_at	SPC25	< 0.001	–3.49
49	211071_s_at	MLLT11	< 0.001	–3.49
50	205085_at	ORC1	0.003	–3.48

The top 50 genes significantly down-regulated by docetaxel when compared with DMSO control in HCC1806.

Supplementary Table 4: Gene microarray results

	Transcript Cluster ID	Gene Symbol	ANOVA *p*-value	Fold Change (linear)
1	232546_at	TP73	0.004	–3.3
2	205393_s_at	CHEK1	< 0.001	–2.77
3	211804_s_at	CDK2	0.004	–2.75
4	1555772_a_at	CDC25A	0.001	–2.73
5	231534_at	CDK1	0.002	–2.72
6	204531_s_at	BRCA1	0.002	–2.7
7	205733_at	BLM	< 0.001	–2.7
8	204240_s_at	SMC2	< 0.001	–2.58
9	204252_at	CDK2	< 0.001	–2.53
10	213253_at	SMC2	0.002	–2.53
11	219715_s_at	TDP1	0.02	–2.5
12	223545_at	FANCD2	0.005	–2.47
13	203214_x_at	CDK1	0.001	–2.44
14	205024_s_at	RAD51	0.002	–2.41
15	242560_at	FANCD2	0.004	–2.29
16	210559_s_at	CDK1	< 0.001	–2.27
17	209715_at	CBX5	< 0.001	–2.26
18	206106_at	MAPK12	0.002	–2.22
19	203062_s_at	MDC1	< 0.001	–2.19
20	242069_at	CBX5	< 0.001	–2.19
21	205394_at	CHEK1	< 0.001	–2.17
22	210416_s_at	CHEK2	0.006	–2.1
23	211851_x_at	BRCA1	0.002	–2.09
24	209257_s_at	SMC3	< 0.001	–2.05
25	229228_at	CREB5 LOC401317	0.01	2.03
26	202284_s_at	CDKN1A	< 0.001	2.59

Changes in genes of ATM pathway in HCC1806 cells treated by docetaxel compared with DMSO treatment. The result shown above was provided by Ingenuity Pathway Analysis.

Supplementary Table 5: Correlation between CD24 expression and cancer recurrence in nine TNBC patients treated with docetaxel-based chemotherapies

Case #	Neoadjuvant	Adjuvant	Sample collected	CD24 Expression	Recurrence	Sites of Recurrence	Time to Recurrence (months)	Duration of Follow-up (months)
1	Docetaxel, doxorubicin, cyclophosphamide	Docetaxel and cyclophosphamide	Post-neoadjuvant	CD24 0	No			15.8
2	Docetaxel and carboplatin	Docetaxel and carboplatin	Baseline	CD24 1+	No			108.0
3	Docetaxel and carboplatin	Docetaxel and carboplatin	Post-neoadjuvant	CD24 3+	Yes	Neck, liver, lung, bone, brain	29.3	
4	Docetaxel, doxorubicin, cyclophosphamide	Paclitaxel and Capecitabine	Post-neoadjuvant	CD24 3+	Yes	Ipsilateral breast	5	
5	Docetaxel and cyclophosphamide	No	Baseline	CD24 1+	Yes	Axillary lymph node	19.2	
			Post-neoadjuvant	CD24 2+				
6	Docetaxel and cyclophosphamide	No	Post-neoadjuvant	CD24 1+	No			14.3
7	Cisplatin and gemcitabine	Docetaxel and cyclophosphamide	Post-neoadjuvant	CD24 1+	No			51.9
8	Docetaxel and carboplatin	No	Post-neoadjuvant	CD24 3+	Yes	Bone, lung, epidural	6	
9	Docetaxel and cyclophosphamide Carboplatin and gemcitabine	No	Post-neoadjuvant	CD24 3+	Yes	Chest wall	8.5	

Table shows the relationship between CD24 expression of tumors before/after neoadjuvant chemotherapy and cancer recurrence in nine TNBC patients. To further confirm the association between CD24 expression and breast cancer recurrence, we performed immunohistochemical staining of CD24 using tumors from an additional nine TNBC patients treated with taxane-based chemotherapy (neoadjuvant with or without postoperative adjuvant chemotherapy). The tumor samples from these patients were stained for CD24 by a laboratory researcher and read by a pathologist blinded for clinical outcomes of the patients. The results showed that all patients with high CD24 expression (2+ or 3+) before or after neoadjuvant treatment developed recurrent disease (mean time to recurrence 13.7 months) and all patients with low CD24 expression (0 or 1+) had no recurrent disease (mean follow up 47.5 months). Patient #5 whose tumor was 1+ before neoadjuvant treatment but increased to 2+ after taxane-based neoadjuvant treatment, later developed recurrent disease.

Supplementary Table 6: Clinicopathological characteristics of the 94 patients studied in TMA

	Number of patients	
	n	%
**Total number of patients**	94	100.0
**Mean age ± SEM**	52.1 ± 10.1	
**Age range**	26–78	
**Menopausal Status**		
Premenopausal	38	40.4
Menopause	53	56.4
Unknown	3	3.2
**Ethnicity**		
Asian/Pacific Islander	8	8.5
Black	14	14.9
Hispanic/Latino	3	3.2
White	66	70.2
Other	2	2.1
Unknown	1	1.1
**Differentiation grade**		
G1(low)	0	0.0
G2 (moderate)	13	13.8
G3 (high)	81	86.2
**Clinical T-Stage**		
T1	29	30.8
T2	43	45.7
T3	12	12.8
T4	6	6.4
Tx	4	4.3
**Clinical N-Stage**		
N0	60	63.8
N+	30	31.9
Nx	4	4.3
**Baseline L/R recurrence and/or mets**		
L/R only	2	2.1
Mets only	4	4.2
L/R and distant mets	4	4.2
**Chemotherapy**		
Yes	94	100
No	0	0
Unknown	0	0
**Surgery**		
Lumpectomy	49	52.1
Mastectomy	38	40.4
Lumpectomy/Mastectomy	6	6.4
Biopsy	1	1.1
Unknown	0	0

G – Grade, L/R – Local/Regional, Mets – Metastasis.

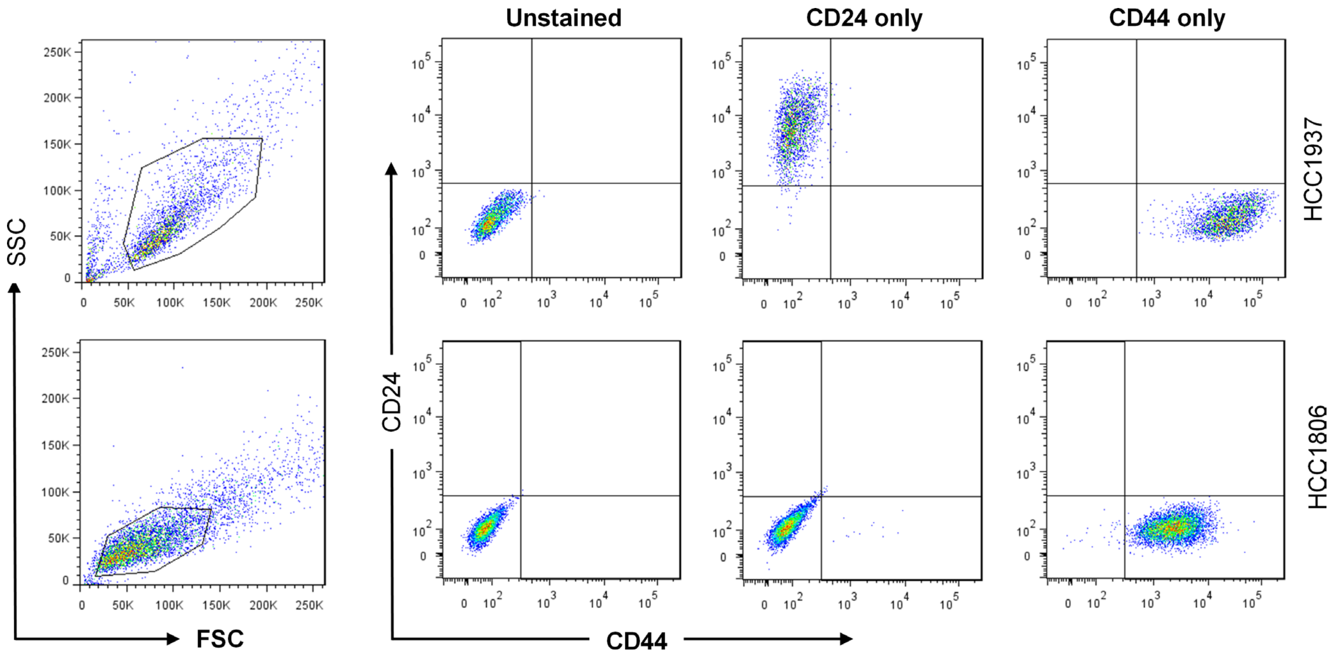

Supplementary Figure 1: Examples for the gating and controls set up for FACS experiments. The untreated controls of two cell lines were used to demonstrate the gating method for FACS analysis. The raw data were first gated using forward scatter (FSC) and side scatter (SSC) to identify viable, single cell events. The cells were then analyzed in bivariate histograms which divides the plots in four quadrants for the four possible combinations: double positive, single positive for each antibody/dye, and negative for both. The gates were set up using the untreated cells for each cell line in each experiment. The same numbers of cells were analyzed in the same set of experiment.

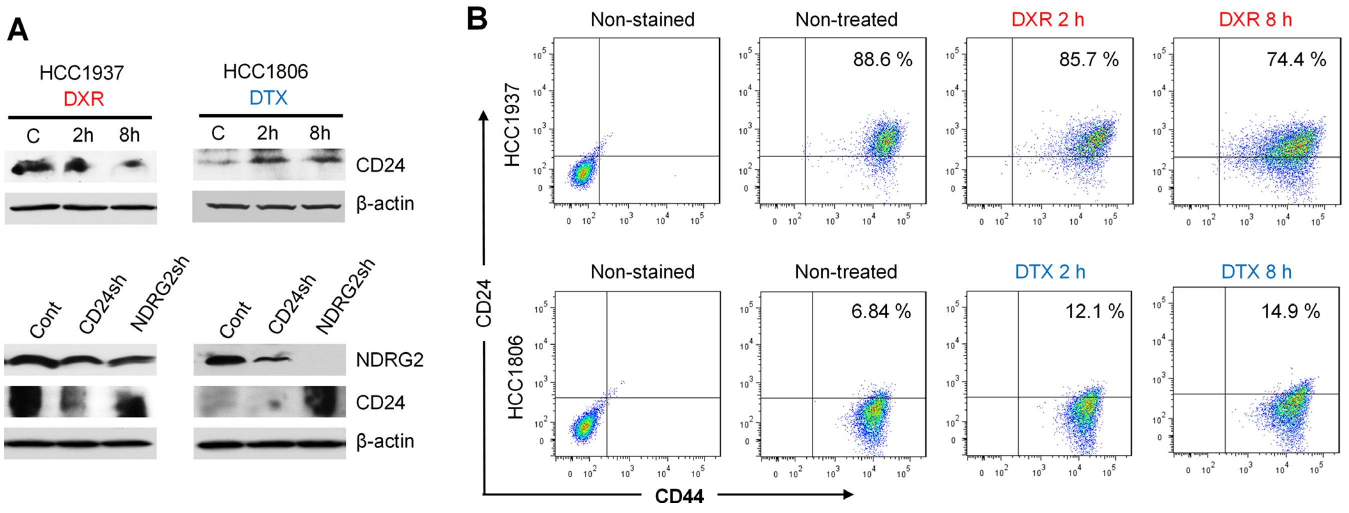

Figure 2: Validation of drug effects on CD24 expression and knockdown efficiency. (**A**) In the upper panel, the cells were treated with 6.4 µM docetaxel (DTX) or 4 µM doxorubicin (DXR) for different time periods, and the cell lysates were then collected for Western immunoblotting. In the lower panel, stable cell lines transfected with different plasmids were compared in Western immunoblot analyses. The NDRG2 knockdown has no significant effect on CD24 expression in HCC1937 because of the high basal level of CD24 of this cell line. Similarly, the CD24 knockdown has no significant effect on CD24 expression in HCC1806 because of its low basal level of CD24. (**B**) Shows the FACS results of two cell lines detected for CD24 and CD44 expression. HCC1937 was treated with 4 µM doxorubicin for 2 h and 8 h. HCC1806 was treated with 6 µM docetaxel for 2 h and 8 h.

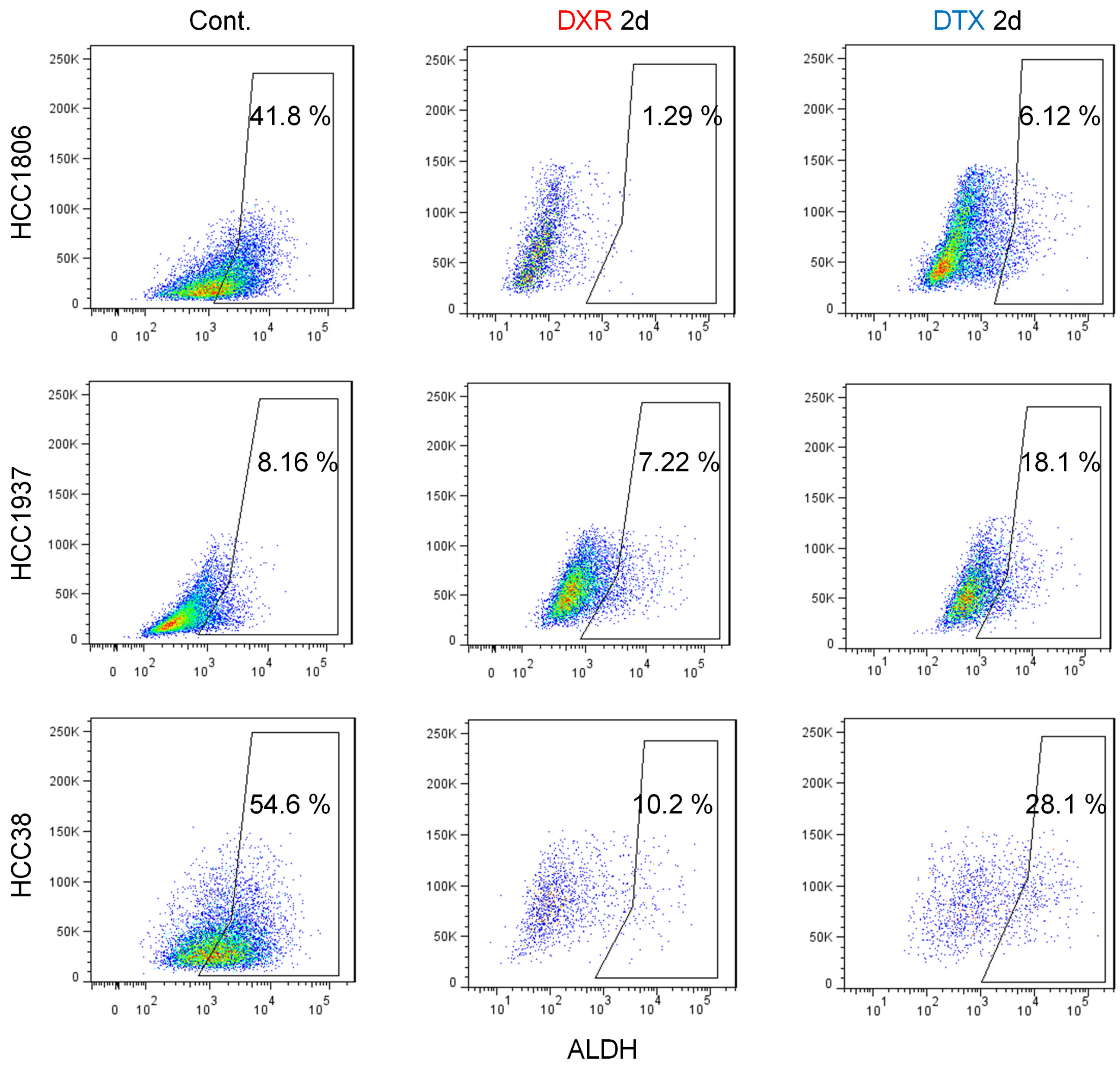

Figure 3: ALDH activity tested in three cells lines. ALDH activity was detected using an ALDEFLUOR assay kit. A specific inhibitor of ALDH, diethylaminobenzaldehyde (DEAB), is used to control for background fluorescence. The cells were treated with 6 μM docetaxel or 4 μM doxorubicin for 2 days before tested in Aldefluor Assay. The results showed that both HCC1806 and HCC38 cells had a large population of ALDH positive cells. In contrast, HCC1937 had a very small group of ALDH positive cells. After doxorubicin treatment, ALDH activity decreased in both HCC1806 and HCC38 cells but had no significant effect in HCC1937 cells. After docetaxel treatment, ALDH activity also decreased in HCC1806 and HCC38 cells but increased in HCC1937 cells.

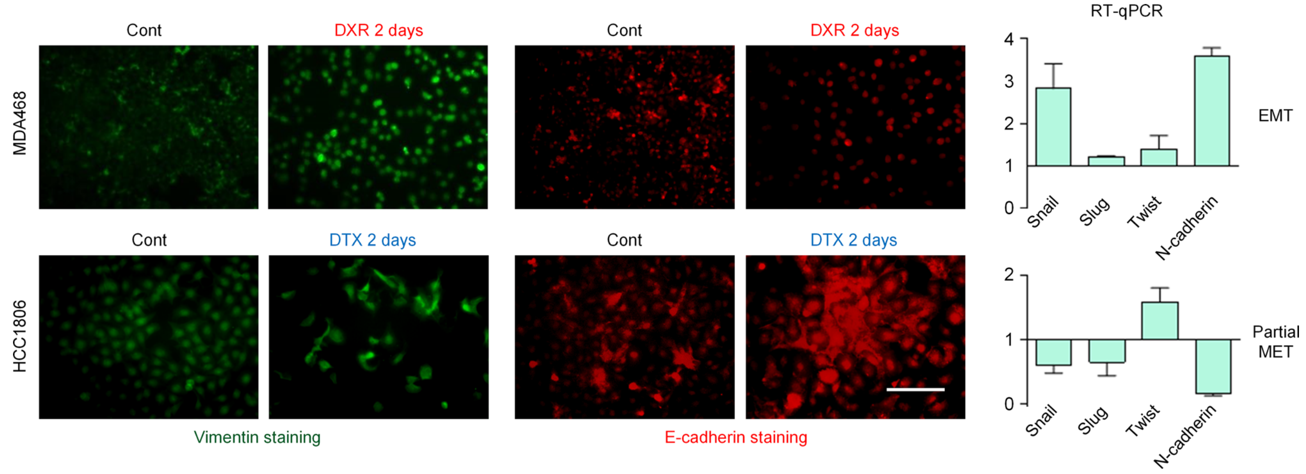

Figure 4: Doxorubicin treatment induced epithelial–mesenchymal transition (EMT), and docetaxel treatment induced partial mesenchymal–epithelial transition (MET) in TNBC cells. The left panel (**A**) shows immunofluorescence staining results, and the right panel (**B**) shows the quantitative real-time PCR results. Both results show doxorubicin-induced EMT changes in MDA-MB-468 cells measured by increased mRNA expression of snail, slug, twist, N-cadherin, with increased vimentin protein expression and decreased E-cadherin protein expression. Docetaxel-induced partial MET changes in HCC1806 cells are shown with decreased mRNA expression of snail, slug and N-cadherin and increased expression of twist mRNA and vimentin and E-cadherin proteins. Error bars represent SEM. Scale bar, 200 µm.

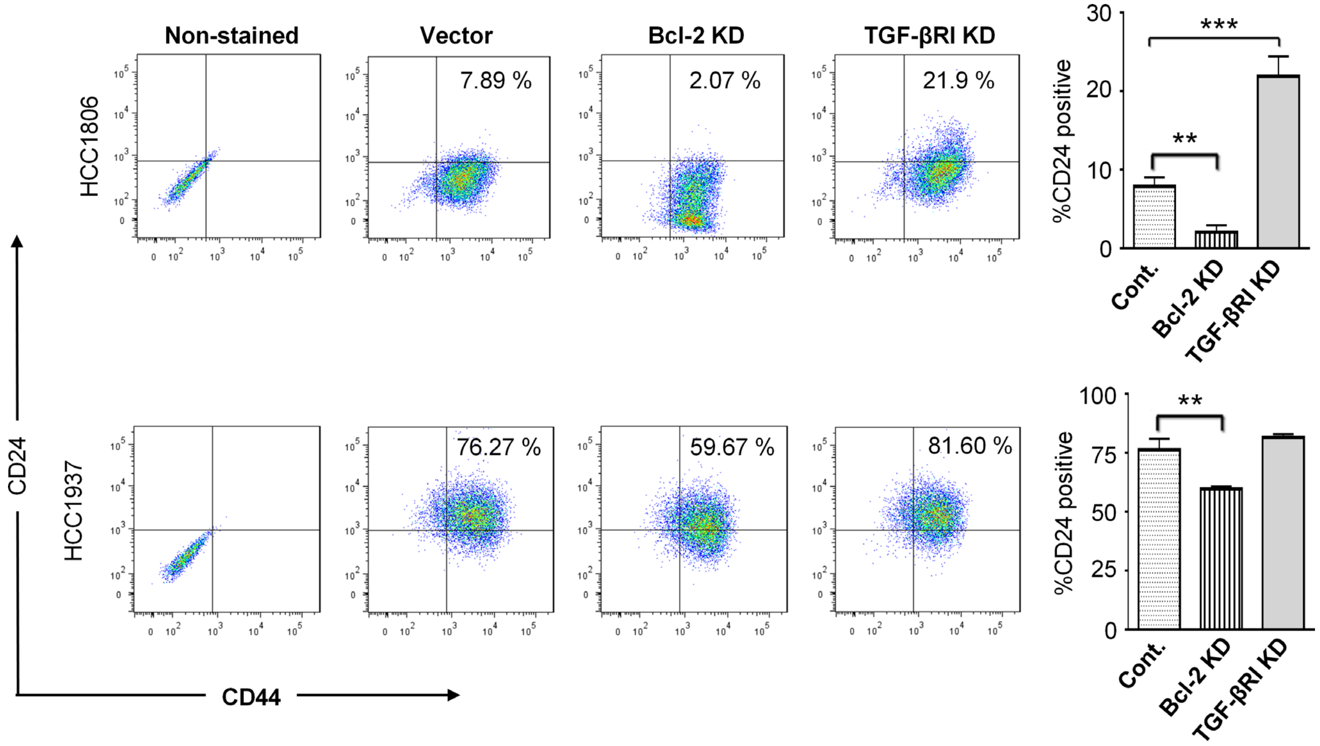

Figure 5: The knockdown effect of Bcl-2 and TGF-βRI in HCC1806 and HCC1937 cells. Results shown by flow cytometry indicated that knockdown of Bcl-2 elicits decreased CD24 expression in HCC1937 and HCC1806 cells; knockdown of TGF-βRI only increased CD24 in HCC1806 but not in HCC 1937 because of its high basal level of CD24. The *p*-values between the compared groups were calculated with unpaired *t*-tests. $^{**}P < 0.01$; $^{***}P < 0.001$.

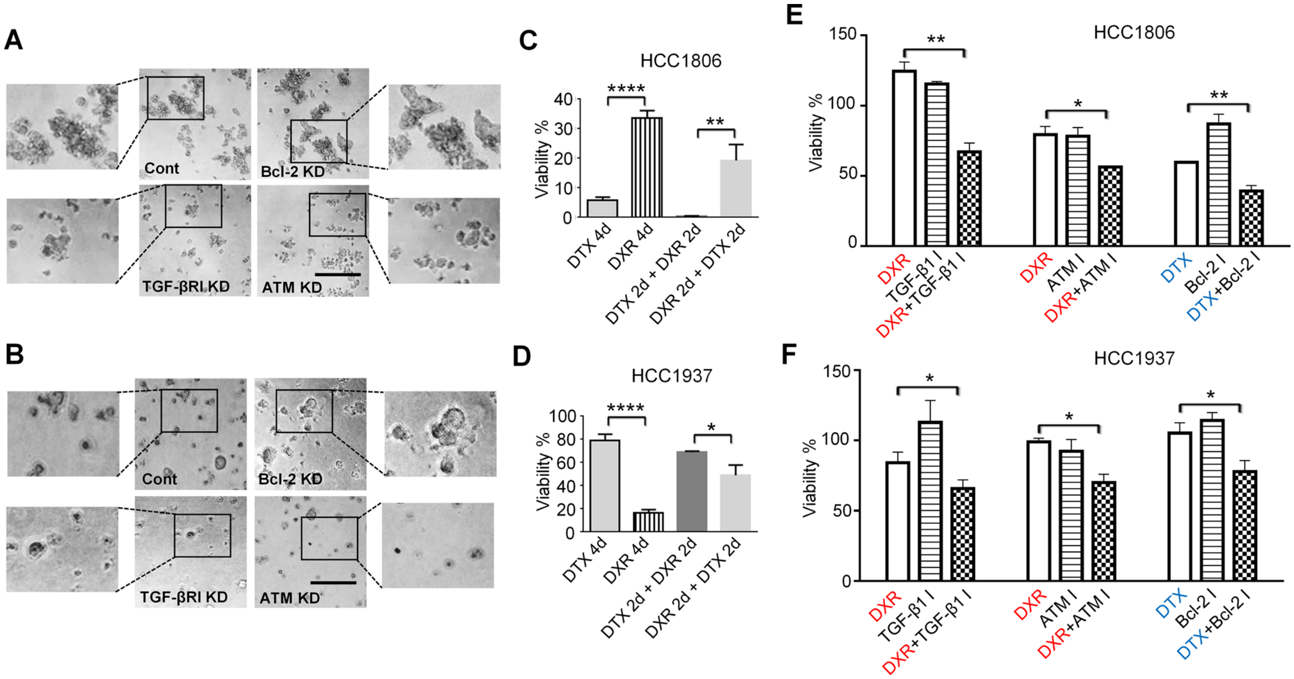

Figure 6: Validation of Bcl-2, TGF-βRI and ATM signaling at the cellular level and drug sensitivity assays with combined treatments. (**A**) Knockdown of Bcl-2 also stimulated self-renewal of HCC1806 cells in suspension culture; while knockdown of TGF-βRI or ATM kinase decreased self-renewal in the same cells. (**B**) Knockdown of Bcl-2 induced differentiation (3D sphere-like structures) of HCC1806 cells while knockdown of TGF-βRI or ATM kinase decreased differentiation of these cells in 3D culture. The cells were cultured on 40 µl matrigel for 7 days with the growth media containing 2% of matrigel. In (**C**) and (**D**), cells were treated with: 500 nM docetaxel (DTX) for 4 days ; 500 nM doxorubicin (DXR) for 4 days; 250 nM DTX for 2 days and then 250 nM DXR for 2 days; 250 nM DXR for 2 days and then 250 nM DTX for 2 days. In (**E**) and (**F**), cells were seeded in matrigel for 6 days in 96-Well plate and treated with the following reagents for 4 days: DMSO as control, 1 µl/well; docetaxel 0.4 µM; doxorubicin 40 nM; Bcl-2 inhibitor G3139 2 µM and ATM inhibitor KU60019 2 µM. (E) and (F) showed that single chemotherapy agent or inhibitor had limited effects on cell viabilities but the combinations of the two drugs and inhibitor dramatically increased the cell killing effects. The *p*-values between the compared groups were calculated with unpaired *t*-tests. $*P < 0.05$; $**P < 0.01$; $****P < 0.0001$. All experiments were done in triplicate. Error bars represent SEM. Scale bar, 200 µm.
